# An Artificial-Intelligence-Driven Predictive Model for Surface Defect Detections in Medical MEMS

**DOI:** 10.3390/s21186141

**Published:** 2021-09-13

**Authors:** Amin Amini, Jamil Kanfoud, Tat-Hean Gan

**Affiliations:** Brunel Innovation Centre, Brunel University London, Uxbridge UB8 3PH, UK; amin.amini@brunel.ac.uk (A.A.); jamil.kanfoud@brunel.ac.uk (J.K.)

**Keywords:** MEMS, defect detection, machine-learning, deep-learning, CNN

## Abstract

With the advancement of miniaturization in electronics and the ubiquity of micro-electro-mechanical systems (MEMS) in different applications including computing, sensing and medical apparatus, the importance of increasing production yields and ensuring the quality standard of products has become an important focus in manufacturing. Hence, the need for high-accuracy and automatic defect detection in the early phases of MEMS production has been recognized. This not only eliminates human interaction in the defect detection process, but also saves raw material and labor required. This research developed an automated defects recognition (ADR) system using a unique plenoptic camera capable of detecting surface defects of MEMS wafers using a machine-learning approach. The developed algorithm could be applied at any stage of the production process detecting defects at both entire MEMS wafer and single component scale. The developed system showed an F1 score of 0.81 U on average for true positive defect detection, with a processing time of 18 s for each image based on 6 validation sample images including 371 labels.

## 1. Introduction

Modern electronic devices, such as smart phones, consumer electronics, healthcare devices, or surveillance and safety assistant systems, combine a huge variety of functions and offer a high level of comfort and functionality in a reduced space. To ensure the high quality of miniaturized products, micro-technology (less than 1 mm in size) offers techniques, tools, and process configurations for the reliable high-volume production of micro-components to achieve a continuous trend of miniaturization and multi-functionalization. In the last decades, micro- and nano-manufacturing and metrology has been driven by micro-electro-mechanical systems (MEMS), where well-established manufacturing methods based on semiconductor technologies are able to produce structures in micro-meter dimensions [1]. In order to produce mechanical micro parts (i.e., metals or polymers), classical manufacturing technologies, such as forming, have to be applied and downscaled from the macro to the micro scale (i.e., micro deep drawing). By downscaling process parameters to the micro scale or vice versa, size defects might occur which lead to unexpected process behavior.

There have been several attempts toward detecting surface defects in steel production as well as in micro-fabricated devices, wafers, and MEMS, based on a non-learning, non-destructive testing (NDT) approach [2,3,4,5]. For instance, in [6], a new technique based on transient infrared thermography in a transmission mode was used to detect a multi-layered MEMS for defect detection. Using finite element analysis (FEA) defect simulation, the research calculated the sample surface temperature differences between defective and healthy regions. It was concluded that using the aforementioned technique, the size of defects could be estimated more consistently using the surface temperature gradient for transmission mode thermography compared to the reflection mode. Nonetheless, this technique would only be able to detect defects such as delamination and voids, with the defects’ size being down to a few hundred microns, if the camera is equipped with a micro-lens that might result in distortion.

In another study, Li et al. [7] applied a high-speed image super-resolution algorithm based on sparse representation for defect detection in MEMS. Unlike traditional super-resolution algorithms that process the whole image at once, the research approach sought to divide the image into several blocks based on different categories and their features and process them individually, achieving significantly higher processing speeds between the high- and low-resolution dictionary pairs. As a result, it was concluded that different defects in MEMS were able to be detected with a significantly lower processing time accepting a slightly lower image quality. The research goal was not to detect the defects in MEMS but to improve the sample images’ detail and quality such that the defects could be more easily illustrated and detected, either by an operator or by an ADR tool.

In [8], a customized deep-learning convolutional neural network (CNN) model was developed to detect defects in the semiconductor wafer manufacturing process. The model was trained to detect four types of defects including circles, clusters, scratches, and spots, and was limited to different defect patterns in a whole wafer. The developed model managed to achieve 84% accuracy in mixed defect detection cases.

In the work carried out by Chen et al. [9], a K-means clustering algorithm was employed for defect detection in the grain surface of silicon wafers. Since the K-means clustering algorithm is prone to noise and as grain surfaces are very noisy, using traditional K-means clustering algorithms would not result in a very high accuracy detection rate. Thus, the authors implemented a pre-processing technique based on morphological operations (closed and open) to reduce the noise. The results demonstrated that the developed algorithm yielded 99.02% accuracy in detecting grain surface defects.

In a study by Shanker and Zhong [10], a template-based vision system for wafer die surface defect inspection was implemented. The system was capable of detecting defects with sizes ranging from 12.7 microns up to around 20 cm. Unlike Devka et al. [8], the researchers considered each die as one part of the whole wafer. A flawless die was used as a template die for the whole wafer to be compared against. The research used image processing techniques to subtract the reference image (healthy die) from every single die in a wafer and, based on the pixel value of the result image, it was possible to determine whether the dies had defects based on the mean square error value, as long as the defect size was within the detectable limit of the algorithm. Moreover, Tien et al. [11] implemented an automatic positioning and wafer detection system based on image processing and fuzzy inference algorithms. A charge-coupled device (CCD) was used, coupled with pre-processing steps, including noise filtering and edge detection, as well as defining the defective template in order to infer its characteristic points to employ it as the reference input for the fuzzy interface. The research adopted a heuristic approach towards detecting, localizing, and classifying defects in a wafer. The results showed a 97.4% average true positive defect detection accuracy for two different defect types (scratches and stains) amongst 153 die samples. In general, the heuristic approach yields higher accuracy when dealing with a limited dataset as it works independently of the number of training datasets. Moreover, heuristic algorithms are very efficient for discrete and simple detections [12]. In defect detection, as long as the detection case is simple enough to be implemented algorithmically, a heuristic approach coupled with some image-processing-based enhancement techniques is adequate. Nonetheless, as the shape and defect characteristics complexity increases, the use of more robust techniques such as machine learning (ML) is needed [12].

In recent years, there have been several attempts to develop ML-based surface defect detection, resulting in more robust and versatile automatic defect detection algorithms [13,14,15,16,17].

Tello et al. [18], conducted research using ML for the recognition of mixed-defect patterns during semiconductor fabrication, a process based on the use of a randomized general regression network (RGRN) model. The research was an extension to the previous work by the authors in which the developed model was capable of detecting 99.8% of the defects in the single-pattern scenario when only one defect category was introduced. Nonetheless, the performance proved to be poor when a wafer had mixed-defect patterns. The paper expanded upon the authors’ previous work in order to increase the detection accuracy of mixed-defect patterns by implementing a deep-structured ML model as well as a novel information gain (IG)-based splitter. Moreover, a spatial filter was applied to reduce model bias during the training phase and to eliminate random noises. The results showed improved model detection accuracy of 86.17% for mixed-defect patterns.

In Xingxing Li et al. [19], a crack detection algorithm was developed based on the Yolov4 target detection method for silicon wafer surface defect detection. Although the model managed to detect over 98% of the true positive defects, it was only targeted towards a single defect detection (surface cracks). Nonetheless, the model was capable of detecting different shapes of cracks on the surface of silicon wafers ranging from short to long.

In another study, Xiaoyan et al. [20] developed a lightweight CNN model dubbed ‘WDD-Net’ for silicon wafer structural defect detection with a very high detection accuracy (99%). The research evaluated the WDD-Net model against two other established CNN models, one based on VGG-16 and the other based on MobileNet-v2, in which the experimental results showed that WDD-Net was five times faster than the 307 KB models, hence the term ‘lightweight’. Nonetheless, the model requires a moderate pre-processing stage since the silicon wafer image needs to be divided into thousands of sub-images for the developed model to work and the localization feature for detected defects is missing.

This research was directed towards the development of an ML model for surface defect detection, based on a proven neural network architecture (CNN), effective both in terms of accuracy and detection speed. As discussed, there are some shortcomings with some of the aforementioned techniques for surface defect detection including the need for computationally expensive pre-processing, and the lack of available real data that requires the development of a side model, a generative adversarial network (GAN), for compensation [21], absence of localization of the defects, and lack of variety in detectable defect types.

## 2. Materials and Methods

For the development of the ADR algorithm, the authors have explored several neural network models and techniques in order to identify the right algorithm that could not only detect and classify different defects but could also be capable of localizing them with region of interest (ROI) bounding boxes. The convolutional neural network (CNN) is a deep learning model used for object detection for single items in different applications including image and video recognition, recommender systems, image classification, medical image analysis, natural language processing, and financial time series [22]. Due to the nature of the MEMS defects, a CNN model could not be used as the core ML algorithm since there were several defects per input image that needed to be localized in addition to being detected and classified. Thus, a CNN derivative model called Region-CNN (R-CNN) was chosen for this task that could deliver all three requirements for the input images, i.e., detection, classification, and localization [23]. The authors decided to use Faster R-CNN, which dramatically improves the overall performance of R-CNN [24]. Faster R-CNN, as the name suggests, is faster compared to R-CNN and Fast R-CNN [25] while achieving the same detection accuracy. The basic concept is to break down the detection of objects into 2 separate phases. In the first phase, regions are identified within the image that are likely to contain the object of interest. The Faster R-CNN then runs on each proposed region in the second phase, and outputs the object category score and the corresponding bounding box coordinates containing the object [24].

CNN, and inherently R-CNN, in order to work with high enough accuracy, require significant amounts of data to be trained [26]. To compensate for this, the authors used a pre-trained publicly available model with a very large number of input images and labels called Common Objects in Context (COCO), and applied transfer learning by training our model on top of the pre-trained one. Using this method, the need for a very significant amount of data for training from scratch was significantly reduced and the model could be tailored to accommodate the specific defects that the task required. COCO is a large-scale object detection, segmentation, and captioning dataset that contains 330 k images (>200 k labelled) of day-to-day objects from persons to chairs and cakes. It contains 1.5 million object instances including 80 object categories, 91 stuff categories, 5 captions per image, and 250,000 people with key points [27].

An ADR neural network model based on TensorFlow Faster R-CNN Inception v2 COCO was developed for this project. There are several base models to choose from, each with advantages and disadvantages. The Faster R-CNN Inception v2 COCO, in particular, has a benchmark detection speed of 58 ms and COCO mAP[^1] of 28 (Table 1). Moreover, higher accuracy models require more computation resources and might not work on mid-range systems and might take a significant amount of time to process one image. Essentially, there is a trade-off between speed and accuracy in industrial environments in which speed is a strong constraint. Thus, Faster R-CNN Inception v2 COCO was chosen as it has very high accuracy and at the same time has a reasonable time delay per input image. CNN-based models have been successfully used previously in surface defect detection [28], making it an ideal model for this research. Additionally, the authors used a residual learning framework (ResNet)-based CNN as its error rate was among the lowest in the ImageNet validation set [29].

**Figure 1:**
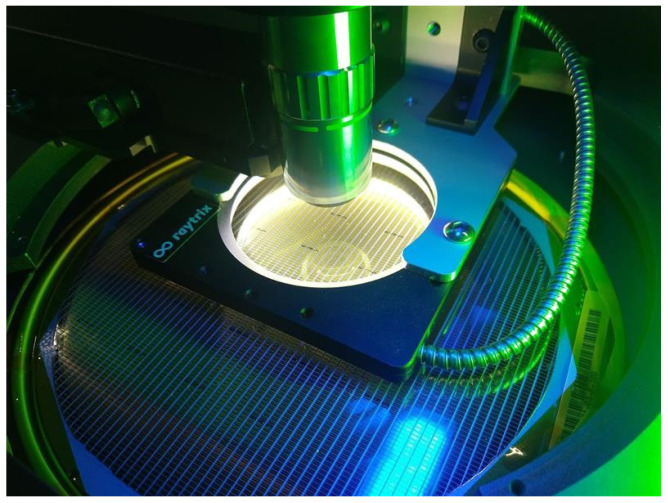
A close-up view of the developed plenoptic camera.

An inspection system consisting of a conventional micro-electrical wafer prober with a plenoptic camera was developed by Raytrix GmbH and retrofitted by aixACCT Systems GmbH (Figure 1). These cameras capture the information of a light ray’s origin in 3D space, via an array of micro-lenses installed closely in front of a conventional photosensitive chip. Once the calibration step of the camera has finished, the imaged object is computationally reconstructed, resulting in a fully focused and 3D depth map image. The chosen cameras are compact and have an extended depth of focus compared to microscope cameras with similar optical properties as the micro-lenses have various focal lengths. The combination of all these advantages renders this technology ideal for MEMS inspection.

### Data Preparation of Plenoptic Images and Training

The images were collected using a conventional micro-electrical wafer probe, retrofitted by aixACCT Systems GmbH with a plenoptic camera developed by Raytrix GmbH. The plenoptic camera captured the information of the light rays’ origin in 3D space, via a micro-lenses array positioned closely in front of a conventional photosensitive chip. Once the calibration process of the camera had been completed, the imaged object was computationally reconstructed, outputting a fully focused image with 3D depth map information.

**Figure 2:**
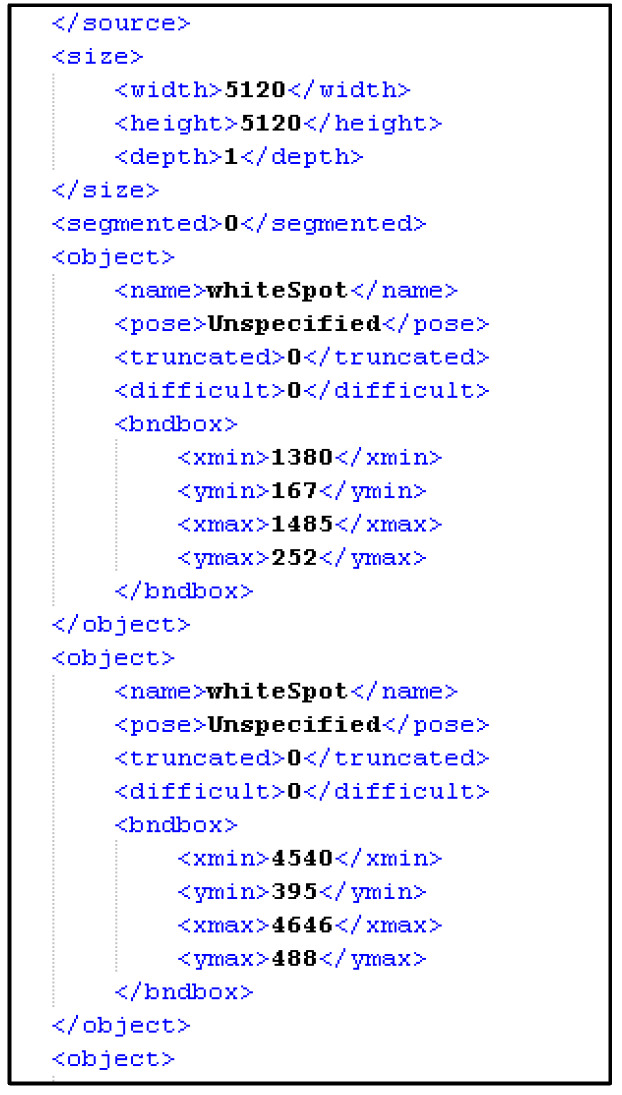
An example of a companion XML file for plenoptic images.

Every image in the dataset was labelled manually by creating a corresponding XML file (Figure 2) for each image containing the X and Y coordinates of every defect in that image alongside the type of that defect. A team of professionals in detecting the end users’ wafer defect types participated in defining and selecting the defects in the sample images. The coordination of these defects was performed manually using software called ‘LabelImg’.

**Figure 3:**
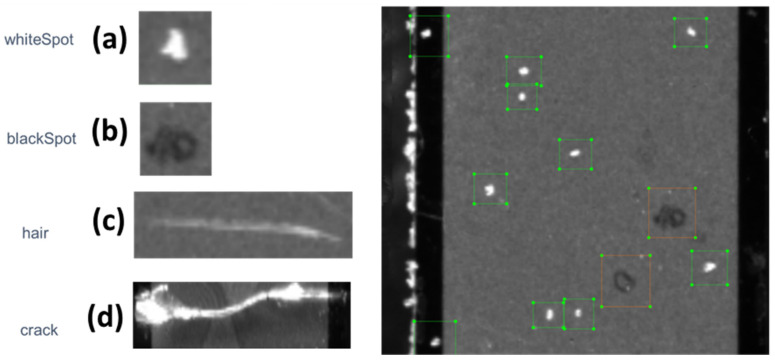
Different defects in plenoptic images. (**a**,**b**) represent small white and black spots, respectively, as a result of the accumulation of dust and dirt. (**c**), as the label suggests, is human hair, whereas (**d**) shows physical cracks on the surface of the specimen due to tension. The right side of the figure represents (**a**,**b**) labelled defects in green and orange rectangles, respectively.

Based on the initial analysis of the input dataset, four different types of defect (Figure 3) were identified among the collected images. Those defect types were the basis of the labelling process.

**Figure 4:**
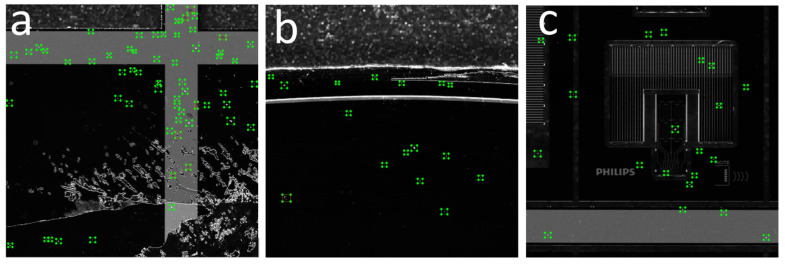
An example of the labelling process. (**a**–**c**) represent parts of a wafer with shatter, upper edge, and middle part, respectively.

Figure 4 shows an example of a labelled image.

At the end of the labelling process, the data were fed into the developed ADR model. Overall, 415 images were used for training and testing. The images were divided into three separate batches: one for training (319), one for testing (90), and one comprising 6 images used for validation purposes (Table 2). In general, the quality of the model depends on four equally important criteria: the quality of the pre-trained model, the quality of the machine learning architecture, the quantity and variability of the training data, and the quality of the labelling. The machine learning architecture and the library were selected after careful analysis of the state of the art as explained above. Although the amount of data is limited by the available dataset, the 415 images represent a suitable quantity of data considering each image had, on average, around 50 defects to be labelled. Among the four criteria, labelling is the only critical factor relying on a manual human process. A total of 20,683 individual labels containing the four different types of defect illustrated in Figure 3 were created. The input image resolution was 5120 × 5120 pixels with an average of ~35 MB in size per image. The number of instances for each defect type varied significantly, with “crack” and “hair” having the lowest occurrence in the entire dataset. The computer used for the training and testing of the developed ADR system was based on a 2 × Intel^®^ Xeon^®^ Gold 6152 CPU (22-Core, 44-Threads, 30.25 MB L3 Cache, up to 3.7 GHz with Intel^®^ Turbo Boost Technology) utilizing an NVIDIA TESLA V100 PCIe 32 GB HBM2, 900 GB/s Bandwidth—DOUBLE-PRECISION: 7 teraFLOPS—SINGLE-PRECISION: 14 teraFLOPS -DEEP LEARNING: 112 teraFLOPS and 640 GB Penta Channel DDR4 at 2666 MHz.

## 3. Results

The plenoptic ADR model based on Faster R-CNN Inception v2 COCO training process took 130 h to be completed for 200,000 epochs. The detection speed, on average, was around 18 s per image and the whole trained model’s total loss was around 0.07, as can be seen from the following table (Table 3).

**Figure 5:**
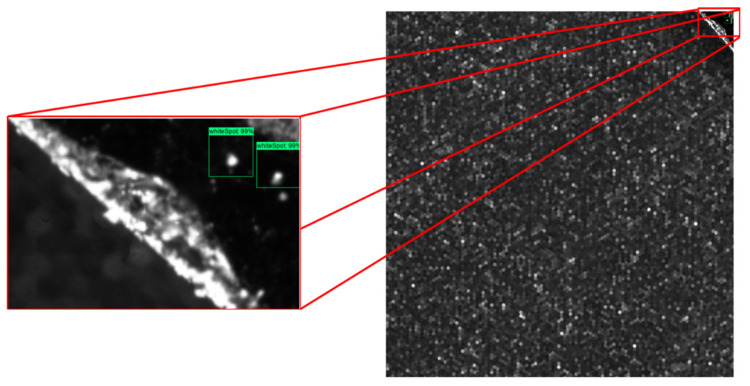
Detected defects of a plenoptic image by the ADR system.

Additionally, a measurement was taken to test and validate the accuracy of the model in terms of the minimum detectable particle and the model’s ability to differentiate the defect from the background. It was concluded that the model had an accuracy along the long semi-axis of 16 um for the detectable minimum particle size. Figure 5 shows three plenoptic images fed to the ADR system for defect detection, classification, and localization. As can be seen, the model detected most of the defects with very high accuracy (>98% confidence level, on average). The developed ADR model was designed so that the minimum confidence level (detection sensitivity) could be manually changed by a user. As the minimum confidence level decreased, the model could detect even more defects, although with lower accuracy.

Table 4 shows the confusion matrix for the developed model against all four defect categories. The results showed that the developed model’s accuracy was high in both average precision and average recall. Nonetheless, the low recall values for “blackSpot” and “hair” defects are due to the fact that the dataset had an imbalanced distribution of the defects’ instances for these two categories.

## 4. Discussion

The development of an ML algorithm based on the Faster R-CNN Inception v2 COCO model to detect and localize surface defects in medical MEMS wafers has proven to be an effective and accurate approach. Even though some research on ADR has been conducted using a heuristic approach with promising results, the complexity of defects and SNR has a direct impact on the accuracy level of these approaches. However, the machine learning approach effectiveness is greatly dependent on the number of samples for training. Correct and accurate data labelling plays a significant role in reducing latency and increasing accuracy. Nevertheless, the overall success rate of a machine learning algorithm with a relatively limited training dataset can be increased by implementing and combining more confidence factors.

Overall, the machine learning approach is ideal for detections that are more sophisticated in terms of shape and color and which require a lot of thresholding, and for variables such as complex defects. However, for simpler cases, such as standard object detection, its disadvantages outweigh its benefits when dealing with very limited datasets, mainly due to its need for a significant amount of system resources (i.e., CPU and memory) to process information beforehand. Moreover, data labelling is a painstaking task and requires a significant amount of time. The developed defect detection model was designed so the minimum detectable confidence level could be manually changed by a user and as the minimum detectable confidence level decreases. For instance, the model could detect more defects at the expense of a lower confidence level. The results also showed a statistically significant true-positive detection rate among the four identified categories. The developed model’s score was high, both in average precision and average recall considering the low occurrence frequency of two of the defect categories “blackSpot” and “hair”, which resulted in lower recall values. Overall, the model managed to detect true-positive defects among all four defect categories with 0.81 F1 accuracy on average, with 18 s of processing time per input image.

## 5. Conclusions

This research sought to develop an automated defect recognition (ADR) system and measurement software capable of detecting surface defects of MEMS using a deep learning approach. The developed algorithm could be applied at any stage of production and assembly process for detecting defects at both the entire-MEMS-wafer and single-component scale. The developed system showed an F1 score of 0.81 U on average for true-positive defect detection with the processing time of 18 s for each image based on six validation sample images including 371 labels.

An ADR system was developed including the software and data-processing algorithms for identification and quantification of imperfections in molded parts and assemblies and their relation to part functionality for plenoptic MEMS images. The ADR software was developed to obtain image data from the plenoptic system. A deep learning neural network algorithm was developed based on the Faster R-CNN Inception v2 COCO and using a transfer learning approach. Consequently, the defects’ features were evaluated and labelled and fed into the developed ML model for training. Moreover, the efficacy of the developed ADR system in detecting, localizing, and classifying defects in MEMS was evaluated and tested. Upon the detection of defects, bounding boxes, with the information about the defects, alongside their detection confidence percentage, were added to the input images, which could be sent to a central system for further analysis and monitoring. The research approach proved to be a reliable and high-accuracy method for detecting surface defects in medical MEMS, the results of which could be applied to similar detection scenarios including solar panel cells, micro fractures, etc. The researchers aim to use the research findings to develop a fully automatic sub-surface defect detections system for medical MEMS.

The findings of this study could cut manufacturing costs significantly as it will offer a system with automated knowledge and inspection data-based process feedback that would allow the detection and traceability of faults that may occur in MEMS production, especially for critical applications such as aerospace, space, and healthcare. It could provide technological and competitive advantage in the growing manufacturing and production industry.

## Figures and Tables

**Table 1:** Comparison between different Faster R-CNN models [30].

Model Name	Detection Speed (ms)	COCO mAP[^1]
faster_rcnn_resnet50_coco	89	30
faster_rcnn_inception_v2_coco	58	28
faster_rcnn_inception_resnet_v2_atrous_coco	620	37
faster_rcnn_resnet50_coco	89	30

**Table 2:** The number of images and labels used in training, testing, and validation for the plenoptic ADR system.

Type	Number of Samples	Number of Labels
Training	320	14,745
Testing	89	5567
Validation	10	246
Total	419	20,558

**Table 3:** The Faster R-CNN Inception v2 COCO trained model performance result.

Model Name	Value (What Is the Unit)
TotalLoss	0.07094
Loss/BoxClassifierLoss/classification_loss	0.03278
Loss/BoxClassifierLoss/localization_loss	0.01471
Loss/RPNLoss/localization_loss	0.01361
Loss/RPNLoss/objectness_loss	0.01215
clone_loss	0.05699
regularization_loss	0.01395

**Table 4:** Confusion matrix for the developed ML model based on Faster R-CNN Inception v2 COCO in detection of medical MEMS defects.

Label ID	Category	Precision_@0.5IOU	Recall_@0.5IOU	Number of Occurrence
0	whiteSpot	0.997915	0.986119	19,858
1	blackSpot	1.000000	0.666666667	109
2	crack	0.936170	1.000000	119
3	hair	1.000000	0.111111	597
Average		0.98	0.69	
F1	0.81			
